# The flicker fusion frequency of budgerigars (*Melopsittacus undulatus*) revisited

**DOI:** 10.1007/s00359-016-1130-z

**Published:** 2016-11-11

**Authors:** Jannika E. Boström, Nicola K. Haller, Marina Dimitrova, Anders Ödeen, Almut Kelber

**Affiliations:** 1 Department of Ecology, Uppsala University, Norbyvägen 18D, 75236 Uppsala, Sweden; 2 Department of Biology, Lund University, Sölvegatan 35, 22362 Lund, Sweden

**Keywords:** Visual ecology, Avian vision, Temporal resolution, Flicker fusion frequency, Psittaciformes

## Abstract

While color vision and spatial resolution have been studied in many bird species, less is known about the temporal aspects of bird vision. High temporal resolution has been described in three species of passerines but it is unknown whether this is specific to passerines, to small actively flying birds, to insectivores or to birds living in bright habitats. Temporal resolution of vision is commonly tested by determining the flicker fusion frequency (FFF), at which the eye can no longer distinguish a flickering light from a constant light of equal intensity at different luminances. Using a food reward, we trained the birds to discriminate a constant light from a flickering light, at four different luminances between 750 and 7500 cd/m^2^. The highest FFF found in one bird at 3500 cd/m^2^ was 93 Hz. Three birds had higher FFF (82 Hz) at 7500 cd/m^2^ than at 3500 cd/m^2^. Six human subjects had lower FFF than the birds at 1500 but similar FFF at 750 cd/m^2^. These results indicate that high temporal resolution is not a common trait for all small and active birds living in bright light habitats. Whether it is typical for passerines or for insectivorous birds remains to be tested.

## Introduction

Most species of birds rely on vision for many important behaviors, and it is no surprise that some species have evolved vision of extremely high acuity. Birds have excellent color vision abilities (e.g., Martin and Osorio 2008; Olsson et al. 2015), some species of acciptriform raptors have the highest spatial acuities known in any animal (Fischer 1969; Reymond 1985), and pigeons (Dodt and Wirth 1953) as well as blue tits and Old World flycatchers (Boström et al. 2016) see the world with a temporal resolution unsurpassed by any other vertebrate. The evolutionary benefit from maximizing spectral, spatial or temporal acuity may be found in the ecology of birds.

While a lot of efforts has been devoted to studies on color vision (for references see Martin and Osorio 2008; Hart and Hunt 2007; Olsson et al. 2015) and spatial resolution (e.g., Ghim and Hodos 2006; Harmening et al. 2009; Lind and Kelber 2011; Lind et al. 2012 and references therein) of birds, our knowledge about avian temporal visual acuity is still quite limited (cf. Dodt and Wirth 1953; Greenwood et al. 2004; Boström et al. 2016), and there are very few clues in the literature as to how widespread ultra-rapid vision is among birds.

As the highest temporal resolution has been found in three small species of insectivorous passerines (Boström et al. 2016), we suggest four possible hypotheses that can be tested: (a) Very high temporal resolution may be a synapomorphy of Passeriformes. (b) It may be a common feature for small fast moving birds with high metabolic rates. Animals that fly fast and control flight by visual cues require high temporal resolution. This has been demonstrated in insect species such as flies and dragonflies (Vogel 1957; Ruck 1958, 1961). Moreover, it has recently been hypothesized that vertebrates with small body size and high metabolic rates should have high temporal acuity (Healy et al. 2013). (c) High temporal acuity could also be closely related to a diurnal activity cycle and a life in very bright habitats. This is suggested by the fact that temporal resolution generally is higher in brighter light levels, and for cone-based as compared to rod-based vision (e.g., Lisney et al. 2011). Finally (d), lifestyles that require accurate tracking of rapid motion may select for high temporal resolution. If so, then raptors and insectivorous birds catching fast flying prey in flight and forest birds speeding through canopies should have the highest resolution.

Similar hypotheses have been formulated for insects already more than 50 years ago. Autrum (1949), and Autrum and Stoecker (1950) studied fly and bee vision and, comparing their results with those obtained in slower moving insects concluded that only fast flying insects have high temporal resolution. Their behavioral results were confirmed by their own and later (e.g. Laughlin and Weckström 1993) electrophysiological results showing that diurnal, fast flying species have faster phototransduction and potassium channels in the photoreceptors than slowly flying and nocturnal species.

Temporal resolution is commonly assessed by measuring flicker fusion frequencies (FFFs), the frequencies at which temporally alternating light–dark stimuli cease to appear as flickering and are perceived as continuous by the observer. FFF increases logarithmically with the luminance of the flickering light, according to the Ferry-Porter Law (Brown 1965), up to a peak value. It is, therefore, common to determine this critical flicker fusion frequency (CFF), the maximal FFF at any luminance, which is the most coherent value for the comparison between species (e.g., Ordy and Samorajski 1968; Jenssen and Swenson 1974; Healy et al. 2013).

Flicker fusion frequency can be estimated both electrophysiologically by electroretinography (ERG), and behaviorally. ERGs are likely to estimate higher FFFs since they measure neuronal transmission at an early processing stage in the retina, and do not take temporal summation, that may occur at later stages into account (D’Eath 1998; Lisney et al. 2012).

Behavioral studies take into account the complete visual pathway of the tested individual and provide an estimate of what the animal perceives. Early studies on birds and insects used an optomotor response to moving gratings to behaviorally determine CFF, however, it is not fully clear that their results are not limited by spatial resolution (Crozier and Wolf 1941, 1944; Autrum and Stoecker 1950). Newer studies use operant conditioning with stationary stimuli (e.g. Ginsburg and Nilsson 1971; Lisney et al. 2011). For those few species of mammals and birds, in which both ERGs and behavioral tests have been performed, higher flicker fusion frequencies have been documented with ERG (Lisney et al. 2012 and references therein).

Behavioral studies have documented the highest CFF among vertebrates in birds. Three species of small, insectivorous passerines—blue tit (*Cyanistes caeruleus*), collared flycatcher (*Ficedula albicollis*) and pied flycatcher (*F. hypoleuca*)—were discriminated light flickering with up to 130–145 Hz from a continuous light, at a luminance of 1500 cd/m^2^ (Boström et al. 2016). For comparison, humans can only detect flicker at much lower frequencies, around 50-60 Hz (Brundett 1974), as can most other non-avian vertebrates, although rhesus monkeys can reach at least 95 Hz (Schumake et al. 1968). Comparable behavioral studies with stationary flickering stimuli are rare in birds. Several studies have determined FFFs in chickens, with slightly variable results (71.5 Hz at 100 cd/m^2^, Jarvis et al. 2002; 74 Hz at 800 cd/m^2^, Rubene et al. 2010) but only one individual reached the CFF (100 Hz in one bird, and 87 Hz on average for 15 birds, at 1375 cd/m^2^, Lisney et al. 2011). An older study on budgerigars used a similar technique but very low light intensities (Ginsburg and Nilsson 1971) and found the highest FFF of 74.4 Hz in one of two tested birds at 17 cd/m^2^, a light level comparable to sunrise or sunset (Lind and Kelber 2009).

ERG studies have rarely used very bright light stimuli, and only in three species of birds reached a point close to CFF: between 45 and 70 Hz in owls (*Asio flammeus*, Bornschein and Tansley 1961; *Athene noctula*, Porciatti et al. 1989), up to 119 Hz in domesticated hens (*Gallus gallus domesticus*, Lisney et al. 2011, 2012) and 143 Hz in pigeons (*Columba livia*, Dodt and Wirth 1953). Although CFF of pigeons is *en par* with the passerines, and the hen CFF is not far below, these CFFs that were determined with ERG recordings are not directly comparable to the behaviorally determined results.

With this lack of data, it is impossible to decide which of our four hypotheses may account for the extremely high temporal resolution found in the passerines. In this study, we have behaviorally tested FFF as a measure of temporal resolution in the budgerigar (*Melopsittacus undulatus*) with the aim to shed new light on the four different hypotheses presented above.

Budgerigars are suited to assess whether very high CFF is common and limited to passerines (a), since they belong to Psittaciformes, a phylogenetic sister group to Passeriformes (Hackett et al. 2008; Jarvis et al. 2014). Budgerigars are small, actively flying, exclusively diurnal birds with relatively high metabolic rates (Weathers and Schoenbaechler 1976) but unlike blue tits and Old World flycatchers they are granivores and do not live in woods but in open landscapes, allowing us to disentangle hypotheses (b: high metabolic rates—high CFF), (c: diurnal lifestyle—high CFF) and (d: insectivory and/or forest life—high CFF).

We also wanted to estimate temporal acuity in budgerigars because they are the third most common pet bird worldwide (Perrins 2003). Pet birds are generally kept indoors, mainly in artificial light. Incandescent light bulbs, which have been very common and suitable for avian husbandry, are being phased out worldwide due to their poor energy efficiency (US Congress and Natural Resources 2005; European Commission 2009) and replaced by various types of fluorescent or light-emitting diod (LED) lamps. In areas where alternating current (AC) power supply has a 50 Hz frequency, many of these lamps flicker at 100 Hz (accordingly, in a number of American countries, 120 Hz). Although this flicker frequency is too high to be perceived by humans, it may induce general stress and impaired welfare in birds with higher FFFs (e.g., Nuboer et al. 1992; Prescott et al. 2003), as has been shown in several studies on starlings (*Sturnus vulgaris*) (Maddocks et al. 2001; Greenwood et al. 2004; Smith et al. 2005; Evans et al. 2006, 2012). If flicker fusion frequencies in budgerigars supersede those of fluorescent and LED lamps, it may spell welfare problems for many pet birds.

## Methods

### Study species

The budgerigar (*Melospittacus undulatus*) is an Australian granivorous parrot in the order Psittaciformes. Budgerigars are nomadic and normally live in small flocks in open grasslands, scrublands and woodlands in dry inland areas, but under favorable conditions they can form large flocks of up to several thousand individuals (Perrins 2003).

### Holding conditions

We experimented on five budgerigars (one female and four males) aged between six months and seven years, who all had previous experience of behavioral trials. The birds were kept in pairs in cages measuring 80 × 45 × 70 cm. They were fed mixed seeds ad libitum, supplemented with minerals, lettuce and carrots and were given unlimited access to water throughout the experimental period. On training and test days (normally five days per week) the birds were only fed seeds during experimental sessions, twice a day, but still received vegetables in their holding cages.

### Experimental setup

The experiments were performed in a Skinner box measuring 100 × 60 cm in area and 72 cm in height, placed in the same room as the holding cages so that the birds could still hear each other, but separated from the other cages by black, unreflective fabric. The Skinner box was illuminated evenly from above using UV LEDs (LZ4-00U600, LED Engin Inc., San Jose, CA, USA) and white LEDs (LZC-00NW40, LED Engin Inc.) powered by a 175 Watt dual power supply (CPX200, Thurlby Thandar instruments Ltd., Huntingdon, England). A calibrated spectroradiometer (AvaSpec-2048 connected to an Avantes CC-UV/VIS cosine corrector; AvaSoft 7.0 computer software; Avantes, Apeldoorn, NL) was used to set the intensities of UV and white LEDs such that the ratio of UV light (<400 nm) and longer wavelength light resembled the ratio in natural daylight (D65). The LEDs were directed upwards and light was reflected into the cage by aluminum foil to distribute the light evenly inside the Skinner box. The illuminance in the Skinner box, the luminance of the background, as well as the light reflected from a white paper on the cage floor were measured using a Hagner ScreenMaster instrument (B. Hagner AB, Solna, Sweden). Cage luminance, measured with a photometer pointing at an angle of 45° downwards to a white paper on the cage floor, is given to allow comparison with earlier studies on budgerigar vision (e.g., Lind and Kelber 2009). The background light was kept considerably darker than the light stimuli, to avoid influences from reflected cage illumination on the stimulus intensities (see Table 1) For two stimulus intensities, we tested different intensities of the background illumination.

**Table 1:** Stimulus and cage illumination for all tests

Stimulus luminance (cd/m^2^)	Background luminance (cd/m^2^)	Cage illuminance (lux)	Cage luminance (cd/m^2^)
750	1.1	30	4.3
1500	2.3	60	9
	5.2	150	20.5
3500	5.3	120	17.5
	11.5	350	45
7200	9	240	35

Stimulus luminance was measured with a photometer pointing towards the stimuli. Background luminance was measured 5 cm above the stimuli using a photometer pointing directly towards the background. Cage illuminance was measured 5 cm above the starting perch of the birds, using a photometer pointing upwards. Cage luminance was measured with a photometer pointing at an angle of 45° downwards to a white paper on the cage floor

The light stimuli were placed 30 cm apart and 30 cm above the floor on one of the short ends of the Skinner box. Under each stimulus a food container with a perch was placed. The food containers, containing the seed mixture, were covered by lids, which could be opened by the experimenter to allow access to the food reward. The birds started each trial from a start perch, 50 cm from the stimuli. They were filmed from behind by a video camera placed on the end of the box opposite to the stimuli. The video image was observed by the experimenter on a monitor invisible to the bird.

### Light stimuli

**Fig. 1:**
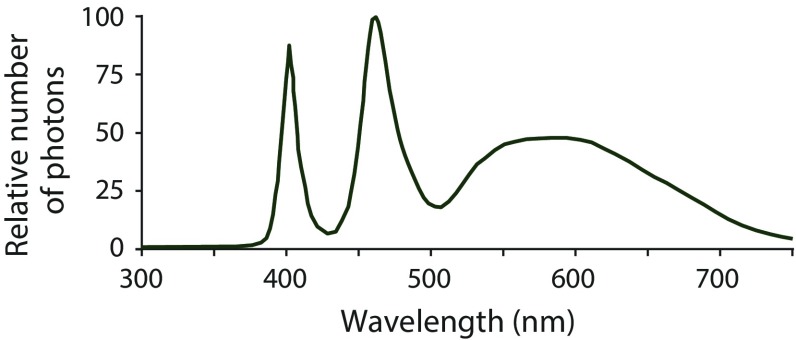
The spectral distribution of the stimulus light used for the experiments

Light stimuli consisted of up to six 5 mm LEDs, both white (Kjell and Company, Malmö, Sweden) and UV (Roithner Laser Technik GmbH, Vienna, Austria), combined and calibrated such that the ratio of UV and long-wavelength light resembled the ratio in natural daylight as perceived by the birds. This was confirmed through spectroradiometer measurements (Fig. 1; AvaSpec-2048 connected to an Avantes CC-UV/VIS cosine corrector; AvaSoft 7.0 computer software; Avantes, Apeldoorn, NL). The LEDs were placed inside aluminum tubes with 18 mm inner diameter, and a UV-transparent Perspex panel was attached at the opposite end. The luminance of the lamps could be lowered using 25, 50 and 75% neutral density and 75% transmission diffusion filters (Lee Filters, Andover, UK) and aluminum tubes of different lengths (80 or 120 mm). The frequency and square-wave shape (100% modulation) of the light–dark cycles of light stimuli were controlled by function generators (2 MHz, GW Instek, Suzhou, China and 3 MHz, TENMA, Taiwan).

### Experimental procedure

The experiments were conducted during July–August 2014. Using operant conditioning and positive reinforcement, we trained the budgerigars to fly from the start perch to the perch in front of a perceptually constant (flicker frequency 20 kHz) lamp, where they received a food reward; flying to a simultaneously presented lamp flickering at 40 Hz was not rewarded. When a bird had learnt the task and repeatedly flew to the rewarded stimulus 4 out of 5 times, the frequency of the unrewarded stimulus, the visibly flickering light was increased in steps of 10 Hz until the animal could no longer distinguish between the stimuli and chose randomly. At this stage the frequency of the unrewarded stimulus was decreased to the last frequency that the bird could discriminate from the perceptually constant rewarded stimulus, and the bird was retested. If the bird chose correctly, the frequency of the unrewarded stimulus was increased again in steps of 10 Hz, and the same procedure was repeated upon each incorrect choice. At higher frequencies, we used steps of 5 Hz, and finally 1 Hz, until the flicker fusion frequency was reached. To determine the flicker fusion frequency, the bird was required to successfully discriminate it in two test series, in total choosing the rewarded stimulus a minimum 8 out of 10 times when the unrewarded stimulus flickered at that specific frequency. The procedure was repeated at four different stimulus luminances (750, 1500, 3500 and 7200 cd/m^2^), and the birds were trained and tested individually at all light intensities in random order.

For comparison, and to verify the setup, we tested six human subjects aged 25–68 years in the same experiment at the luminances 750 and 3500 cd/m^2^. The distance between eyes and stimuli was slightly larger for humans (55 cm) than for the birds (50 cm), due to technical reasons. All human subjects wore UV-blocking protection glasses (UVEX Safety Group GmbH & Co. KG., Fürth, Germany) during the tests and were not given any reward for correct choices.

We tested whether the different background illumination intensities (in tests with 1500 and 3500 cd/m^2^) had an influence on the results, using a mixed model, with a random intercept for ‘bird id’, written in the open source software r.

## Results

**Fig. 2:**
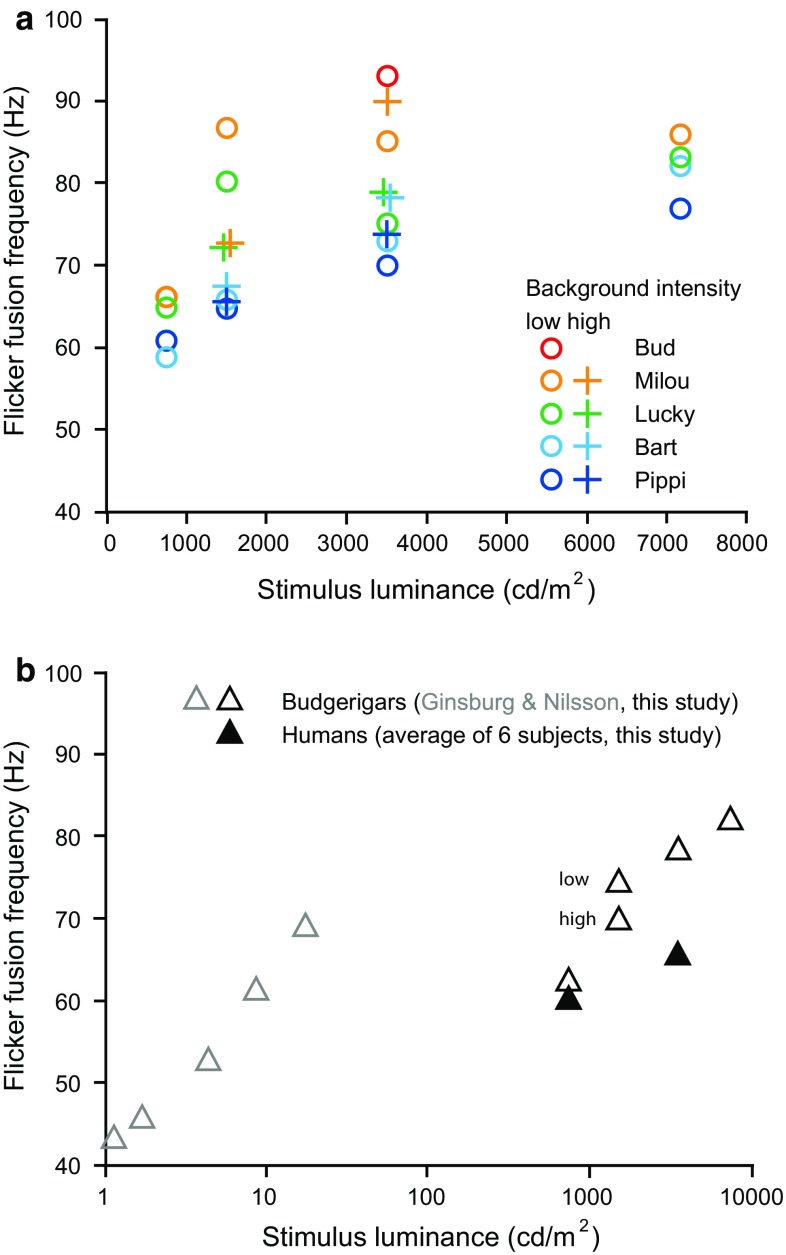
**a** Flicker fusion frequencies of all five birds (Bud, Lucky, Bart, Pippi, Milou) in tests with four luminances. *Open circles*: results with low background intensities; *crosses* results with higher background intensities (see Table 1). **b** Average FFF of budgerigars. Note that the luminance scale here is logarithmic. *Gray open triangles* show the results obtained with two birds in experiments performed and published by Ginsburg and Nilsson (1971). *Black open triangles* show averages obtained with the birds (except Bud, whose data are excluded because he only cooperated in one test) tested in our study (results with low and high background luminances are given as separate points when the results were statistically different). *Filled triangles* show average values obtained with six human subjects under the same conditions

Four of the five budgerigars successfully participated in the experiments at all four light intensities, whereas one male (Bud) only completed the experiment at 3500 cd/m^2^ and then ceased cooperating. For the stimulus luminances 1500 and 3500 cd/m^2^ the trials were performed using two different background intensities (see Table 1). We found a small but significant effect of background intensity on FFF (open circles and crosses in Fig. 2a) in the trials with 1500 cd/m^2^ (general linear model, *z* = 1.752, *p* = 0.24), but not in trials with 3500 cd/m^2^ (general linear model, *z* = 5.356, *p* < 0.001).

One of the birds (Milou) had his highest FFF at 3500 cd/m^2^, whereas for the other three (Lucky, Bart and Pippi) the FFF was slightly higher at 7200 cd/m^2^ than at 3500 cd/m^2^ (Fig. 2a), and their CFFs could, therefore, not be determined.

As the budgerigars were not very motivated to fly in dim light, we did not test them at luminances lower than 750 cd/m^2^. Instead, the data from the study by Ginsburg and Nilsson (1971) are included in Fig. 2b (gray triangles).

For humans the FFFs varied between 55 and 66 Hz at 750 cd/m^2^ and between 57 and 72 Hz at 3500 cd/m^2^ (Fig. 2b). The oldest subject (68 years) had the lowest FFF at both stimulus luminances. This was close to the expected range but somewhat higher than expected given previous results (Brundrett 1974).

## Discussion

### A small desert-living parrot with relatively low FFF

Our results show that the highest flicker fusion frequency, the CFF, of budgerigars occurs at much higher luminances than previously assumed—at least at 3500 or 7200 cd/m^2^, possibly even higher. This is brighter than for any other tested bird species, but follows our expectations since wild budgerigars live in extremely bright, open habitats in the Australian desert, and hence should be adapted to high light intensities. Just like passerines, they have a cone-dominated retina with 2.1 times as many cones as rods (Lind and Kelber 2009). However, the highest FFFs in our experiments, in the frequency range between 77 and 93 Hz, are much lower than CFFs for some fast flying insects, but also birds such as blue tits and Old World flycatchers (Boström et al. 2016) and *en par* with results from domestic chicken (Lisney et al. 2011). Since budgerigars are much closer in size and flight behavior to the passerines than to chickens, our result suggests that small size and airborne agility *per se* does not lead to very high temporal visual resolution. Furthermore, the fact that domestic chicken are descendants from red jungle fowl, living in the dim undergrowth of tropical forests, does not support bright habitats as an explanation to the extreme CFFs found in blue tits and flycatchers.

Our results do, however, support that extreme temporal visual acuity may be a synapomorphic trait for passerines, as there is no conclusive evidence for CFFs in passerines being even nearly as low as in the Psittaciform budgerigar. Crozier and Wolf (1941, 1944) reported 55 Hz CFFs in two passerines, zebrafinch (*Taeniopygia guttata*) and house sparrow (*Passer domesticus*), but because their experimental design recorded optomotor responses, which are limited by both spatial and temporal resolution, the true CFFs may have been underestimated.

It is also possible that lifestyles requiring accurate tracking of rapid motion are driving the evolution of temporal acuity in birds, as has been shown for insects (e.g., Autrum 1949; Autrum and Stoecker 1950; Laughlin and Weckström 1993; Weckström and Laughlin 1995). Budgerigars have different feeding habits from the passerine species tested by Boström et al. (2016). Both pied and collared flycatchers have a diet dominated by insects, while insects form a smaller but significant part of the diet of blue tits (del Hoyo et al. 2006, 2007). Catching flying insects on the wing should exert a high pressure on temporal visual acuity and is likely to have pushed the CFFs of these species, especially in the flycatchers. Budgerigars and chickens, on the other hand, have diets predominated by seeds or slow moving insects, putting less pressure on temporal visual acuity. Another ecological difference between blue tits/flycatchers and budgerigars/chicken are their habitats. Blue tits and flycatchers lead airborne lives in forests, constituting quite complex environments, which may also require high temporal acuities in order for the birds to be able to move and manoeuver fast through the canopies. Red jungle fowl also live in forests, but they move slower and mostly walk around on the ground, a behavior that might not require as high temporal acuities. Budgerigars live in open habitats, facing less risks of colliding with branches and trees, and hence may also perform well with slower vision.

Our experimental design was rather similar to those used for chicken (e.g., Lisney et al. 2011) and passerines (Boström et al. 2016), making our results comparable to the CFFs behaviorally determined in these other bird species. It might, however, be problematic to draw ecological conclusions based on experiments with domesticated budgerigars and chicken, since there are indications that domestication may have had some detrimental effect on the visual system in domestic birds (Lisney et al. 2011; Roth and Lind 2013). Since our test animals had not been caught in the wild, we cannot control for the possibility that loss of visual acuity has occurred in budgerigars during domestication and artificial selection for different color varieties (but see Jeffery and Williams 1994).

### Comparison to Ginsburg and Nilsson (1971)

Our results on FFF in budgerigars in high light intensities appear somewhat low compared to what Ginsburg and Nilsson (1971) found for lower intensities (Fig. 2b). One difference between our study and theirs is that their light stimuli did not include UV light. A study on domestic chicken by Rubene et al. (2010) found that excluding UV light from the stimuli resulted in significantly lower FFF values than if the stimuli contained full spectrum light. On the other hand, judging by visual examination of the graph in Fig. 2b, the FFFs measured by Ginsburg and Nilsson at lower luminances are not lower than expected by our data, if anything they are higher than our curve would suggest by extrapolation.

Other differences between both studies are the applied training and testing regimes. The two budgerigars tested by Ginsburg and Nilsson (1971) were closer to the stimulus, and they were not presented a choice between two stimuli, but trained to peck at a key if the presented light appeared constant. Shortening the distance to the stimulus will increase the size of its image on the retina. In humans this is known to increase FFF, according to the Granit-Harper Law (Granit and Harper 1930). Ginsburg and Nilsson (1971) also started their experiment at high frequencies and decreased the frequency until the bird ceased pecking at the key, whereas our experiment started at lower frequencies which were increased until the bird failed to separate between the two stimuli. Finally, Ginsburg and Nilsson (1971) likely used relatively higher ambient light levels, compared to stimulus luminance, than we did. As we found a difference between FFF with different ambient light levels in the test with 1500 cd/m^2^, this may also have influenced the results. However, we consider the results with high luminance are most relevant for a desert bird, which is only active at daytime, in very bright light.

### Are pet budgerigars seeing the flicker of lamps?

Do budgerigars see the flicker of fluorescent tubes or LED lamps in homes or in pet shops, and does this illumination stress the birds? We aimed at investigating whether the welfare of budgerigars in captivity may be impaired by flickering light, as it should appear flickering to the birds if their FFFs exceed 100–120 Hz. None of the budgerigars in our experiment had FFFs above 100 Hz in any of the tested light intensities, so it is unlikely that they would suffer under fluorescent lights. The European standard for workspace illumination (EN 12464-2:2007) requires a luminance of 500 cd/m^2^ at desks and 100 cd/m^2^ in the general work space. Homes illuminated by fluorescent tubes in bright living rooms may be twice as bright. We also measured the luminance in an office, which is a rather bright environment, lit by fluorescent lamps. Straight under the lamp the luminance was approximately 1000 cd/m^2^ and the luminance dropped quite quickly with increased distance from the lamp. At 750 cd/m^2^ budgerigars and humans did not differ very much in FFF (Fig. 2b), suggesting that even budgerigars exposed to worn fluorescent lamps with flicker frequencies below 100 Hz should probably not detect the flicker more than their human care takers, minimizing the risks of impaired welfare for domestic budgerigars.

Even if budgerigars are unlikely to perceive 100 Hz flicker from artificial lighting it may still cause distress if the retina responds to it. Humans, who normally do not perceive 100 Hz flicker consciously, can still suffer from exposure to it. It can cause headaches, eyestrain, anxiety and changes in eye-saccades (Wilkins et al. 1989), disturb perception of rapid continuous motion (Maddocks et al. 2001) and affect the brain (e.g., Kuller and Laike 1998) or the immune system (Martin 1989). Hence, it is important to study flicker sensitivity in tame birds not only at a cognitive but also at the retinal level, using ERG.

Our study indicates that high temporal resolution is probably not a trait common for all small, active birds, since budgerigars and domestic chicken seem to fall within the same range, whereas vision of the studied passerine species has higher temporal resolution. We consider it more likely that very high temporal resolution of vision may be a synapomorphic trait for passerines or an adaptive trait connected to airborne insectivory or lifestyles of fast flight in complex environments, in a similar ways as has been shown for fast flying insects (see above). Clearly more bird species need to be studied, to resolve this question.

## List of abbreviations

AC: Alternating current
Cd: Candela
FFF: Flicker fusion frequency
CFF: Critical FFF
Hz: Hertz
LED: Light-emitting diod
UV: Ultraviolet
